# The Improvement of Moisture Resistance and Organic Compatibility of SrAl_2_O_4_: Eu^2+^, Dy^3+^ Persistent Phosphors Coated with Silica–Polymer Hybrid Shell

**DOI:** 10.3390/ma13020426

**Published:** 2020-01-16

**Authors:** Lei Lyu, Yuxian Chen, Liting Yu, Rui Li, Liu Zhang, Jianzhong Pei

**Affiliations:** 1 School of Highway, Chang’an University, Xi’an 710064, China; lyulei@chd.edu.cn (L.L.); chenyuxian@chd.edu.cn (Y.C.); yuliting@chd.edu.cn (L.Y.); 2 Shijiazhuang Municipal Design and Research Institute Co., Ltd., 35 Jianshe South Street, Shijiazhuang 050000, China; 2015121190@chd.edu.cn

**Keywords:** SrAl_2_O_4_ persistent phosphors, silica–polymer hybrid shell, moisture resistance

## Abstract

The existing road surface marking with poor visibility at night results in traffic safety hazards in insufficient lighting roads. This study aims to prepare the dedicated aluminate-based persistent phosphors considering the integrated pavement environment, as the first step to achieve the durable luminescent road surface marking. SrAl_2_O_4_: Eu^2+^, Dy^3+^ persistent phosphors coated with silica–polymer hybrid shell were prepared by chemical precipitation and sol-gel method to improve moisture resistance and organic compatibility. The optimum silane coupling agent type and dosage, the surfactant dosage, the optimum sodium silicate dosage, and the coating reaction time in silica shell and polymer shell coating were studied based on the moisture resistance test. The silica–polymer hybrid shell coating balances the organic compatibility and thermal stability as compared to the silica or polymer shell coating in the oil absorption test and thermogravimetric analysis. Ex-Em Spectra, XRD, and SEM method were used to characterize the persistent phosphors, indicating the preparation does not destroy the persistent phosphors. The outstanding durable properties of SrAl_2_O_4_: Eu^2+^, Dy^3+^ persistent phosphors coated with silica–polymer hybrid shell as shown in this research is crucial for its potential application in waterborne luminescent coatings of road surface marking.

## 1. Introduction

The road surface marking is an important transportation infrastructure to convey various official information, especially to guide traffic on the channelized traffic roads [1,2,3]. Room temperature solvent paint, hot melt paint, heating solvent paint, and water-based paint are the four mainstream types of road surface marking paints. Due to the slow construction speed and serious environment pollution, room temperature solvent paint and hot melt paint have been gradually phased out [4,5]. Besides, the road surface marking produced by above method has poor visibility at night, resulting in traffic safety hazards in insufficient lighting roads.

Energy-storing luminescent materials are alternative road coatings to provide enhanced visibility by self-illuminating. They convert absorbed external energy into fluorescence when excited, relying on their own lattice defects to generate energy-level transitions. The energy storing luminescent materials are classified into sulfides, aluminates and aluminum silicon salts. Aluminate-based persistent phosphors are the most widely used and studied luminescent material for its high quanta efficiency, long after-glow life, and good chemical stability [6]. The SrAl_2_O_4_: Eu^2+^, Dy^3+^ crystalizes in a monoclinic system and consists of rings formed by six-corner-sharing oxide aluminate tetrahedra, of which two Sr^2+^ ions (*r* = 126 pm in VIII coordination) are replaced by Eu^2+^ (*r* = 125 pm) and Dy^3+^ (*r* = 97 pm) respectively [7]. The persistent time of SrAl_2_O_4_: Eu^2+^, Dy^3+^ with a strong emission centered at 520 nm (green) was found to be longer than 16 h [8]. However, the luminescent structure of aluminate-based persistent phosphors is gradually damaged at 86.5 °C due to the thermal quenching.

Many researchers have demonstrated the possibility of using aluminate-based persistent phosphors in waterborne paints for road surface markings based on laboratory and field tests [9,10,11]. OssN329 road in the Netherlands is the first road achieving the achieve the self-luminescence of road surface markings using aluminate-based persistent phosphors in water-based paint. However, the road surface markings lost the luminous function in less than 14 days were caused by the rainfall [12]. Literatures also suggest that poor moisture resistance and organic compatibility is the challenge for the aluminate-based persistent phosphor to be widely used in waterborne paints of road surface marking [13,14,15]. Furthermore, little research focused on the thermal stability of the aluminate-based persistent phosphors used in the road surface markings. The highest temperature of pavement surface is over 60 °C, leading to the potential damage to the aluminate-based persistent phosphors [16]. There has also not been much experience of developing the dedicated durable aluminate-based persistent phosphors considering the integrated pavement environment.

The surface coating modification of aluminate-based persistent phosphors is a widely used method to enhance the moisture resistance and the compatibility with organic solvents of waterborne paints. The surface coating modification includes organic coating and inorganic coating. Inorganic coating refers to coating silica, metal oxides, or metal halides on the surface of persistent phosphors by sol-gel method, liquid phase precipitation method and high-temperature solid-state method. Sodium silicate, ethyl orthosilicate and alumina are widely used inorganic coating agents, which has been proved to improve the moisture resistance of coated luminescent materials [17,18,19]. In organic coating, the organic modifier covers the surface of persistent phosphors by forming chemical bonds or electrostatic adsorption to improve the organic compatibility [20]. However, single inorganic or organic single shell coating could not balance the moisture resistance and organic compatibility of aluminate-based persistent phosphors.

**Figure 1:**
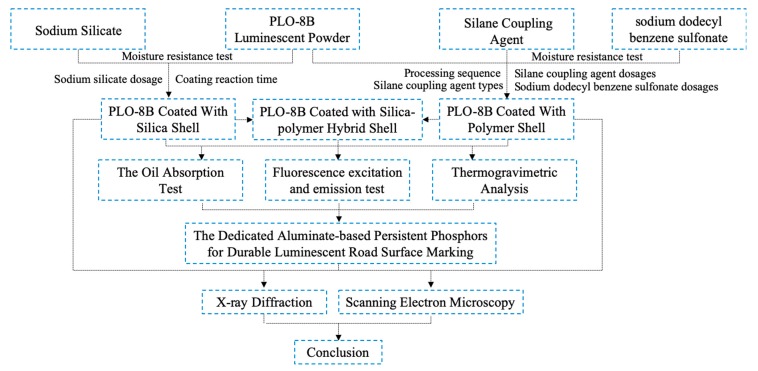
Flow chart of test method.

In this paper, SrAl_2_O_4_: Eu^2+^, Dy^3+^ persistent phosphors were subject to the silica shell coating, polymer shell coating, and the silica–polymer hybrid shell coating. Coating properties were evaluated based on the moisture resistance, organic compatibility, thermal stability, luminosity, and microstructure characterization to explore the optimum composition as shown in Figure 1. The objective of this study was to prepare the dedicated aluminate-based persistent phosphors considering the integrated pavement environment, as the first step to achieve the durable luminescent road surface marking. The simultaneous use of inorganic and organic coating combines their advantages on the moisture resistance and organic compatibility to achieve the balanced design. The moisture resistance and thermal stability of SrAl_2_O_4_: Eu^2+^, Dy^3+^ persistent phosphors were comprehensively evaluated based on the integrated pavement environment for the first time.

## 2. Experiments

### 2.1. Raw Materials

PLO-8B luminescent powder (SrAl_2_O_4_: Eu^2+^, Dy^3+^ persistent phosphors) was purchased from Luming Technology Group. The silane coupling agent, anhydrous ethanol, acrylic monomer (AA), methyl methacrylate (MMA), ethylene glycol, and sodium silicate were produced by Xi’an Chemical Reagent Factory.

### 2.2. Preparation

**Figure 2:**
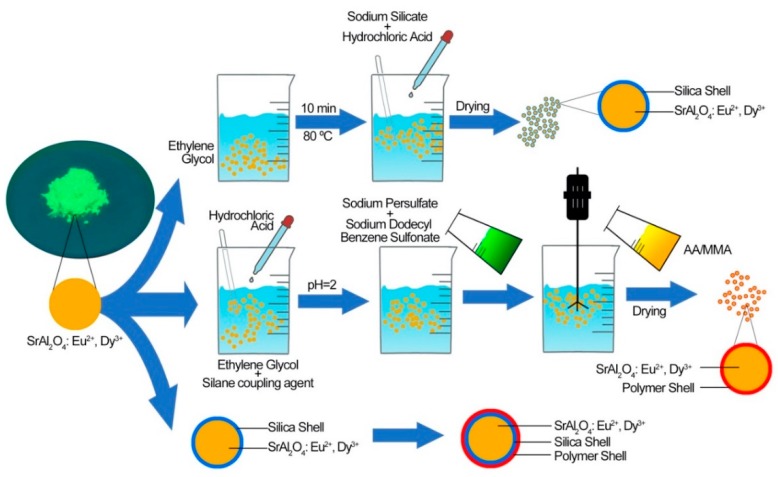
The preparation process of PLO-8B luminescent powder coated with silica shell, polymer shell and silica–polymer hybrid shell.

The PLO-8B luminescent powder coated with silica shell, polymer shell and silica–polymer hybrid shell were prepared to evaluate the effect of compositions on coating properties as shown in Figure 2.

#### 2.2.1. Preparation of Persistent Phosphors Coated with Silica Shell

The PLO-8B luminescent powder was coated with sodium silicate by heterogeneous precipitation method in ethyl alcohol. In the acidic solution, sodium silicate gradually deposits into Si(OH)_4_ colloid as shown in Reaction 1, and bonding with the hydroxyl group having high activation energy to form a dense protective shell.

Na_2_SiO_3_ + 2H^+^ + (X − 1)H_2_O → SiO_2_·XH_2_O + 2Na^+^ (1)

In this preparation, ethylene glycol and PLO-8B luminescent powder was dispersed for 10 min by the parallel feed process. The sodium silicate was poured to solution pH = 9 (80 °C) and stirring quickly. Then the mixture was washed, filtered and dried to obtain PLO-8B luminescent powder coated with silica shell. The initial material dosages of the coating with silica shell is shown in Table 1.

#### 2.2.2. Preparation of Persistent Phosphors Coated with Polymer Shell

**Figure 3:**
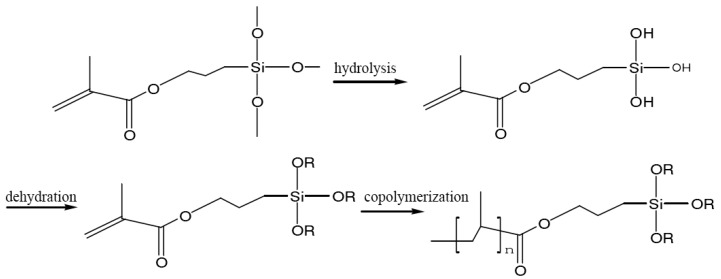
The reaction process of persistent phosphors coated with polymer shell.

Literatures suggested the pretreatment with transparent and well-compatibility silane coupling agent is necessary for persistent phosphors to enhance the modification effect of the organic coating [21]. Due to the alkoxy functional groups of the silane coupling agent produce highly active hydroxyl functional groups after hydrolysis reaction, the first shell was formed on the surface of persistent phosphors. After the pretreatment, the persistent phosphors with a silicon dioxide shell was then coated with the polymer shell from the polymerization reactions of acrylic acid monomer (AA) and methyl methacrylate (MMA) by sol-gel method to achieve the graft modification of the polymer shell as shown in Figure 3 [21].

Specifically, PLO-8B luminescent powder, silane coupling agent and absolute ethanol were mixed in the container. The pH of the solution was adjusted to 2 by adding hydrochloric acid and placed at 80 °C for 4 h. Then, the fully dissolved solution of sodium persulfate and sodium dodecyl benzene sulfonate was added. The mixture of AA/MMA was slowly poured in low speed stirring. After 5 min stationary and removing the upper white suspension, the persistent phosphors coated with polymer shell was obtained by washing with absolute ethanol, filtering and drying. The initial material dosages of the coating with polymer shell is shown in Table 2.

#### 2.2.3. Preparation of Persistent Phosphors Coated with Silica-Polymer Hybrid Shell

Based the former tests, sodium silicate and acrylic acid monomer/methyl methacrylate was used as the inorganic modifier and organic modifier, respectively. The coating process is the same as above. The initial material dosages of the silica–polymer hybrid shell coating is shown in Table 3 [22].

### 2.3. Characterization Method

#### 2.3.1. The Moisture Resistance Test

Improving the moisture resistance of the persistent phosphors is the main purpose of coating, which also deeply affects its service life. Therefore, the effects of different factors in silica or polymer shell on the moisture resistance of the persistent phosphors were investigated.

(2) $${\mathrm{SrAl}}_{2}O_{4}{\;+\;4H}_{2}O\;\to {\;\mathrm{Sr}}^{2+}{+\;2\mathrm{OH}}^{-}{\;+\;2\mathrm{Al}(\mathrm{OH})}_{3}↓$$

As showed in the Reaction (2), SrAl_2_O_4_: Eu^2+^, Dy^3+^ persistent phosphors are destroyed with water, which contributes to the degradation of their luminescent property and the increase of solution pH [23]. Due to the longest average rainfall duration in China being 5.4 hours based on the Chinese meteorological statistics [24,25], pH of the solution after 6 hours was selected to evaluate the moisture resistance. In the test, 0.5 g of luminescent powder was soaked in 40 °C water. The change of pH with time was measured using a digital pH test pen. The growth rate of solution pH (PR) soaked with PLO-8B luminescent powder coated with different shells was calculated to evaluate the improvement of moisture resistance by the following Equation:

$$\mathrm{PR}\;=\;\frac{SP_{f}-SP_{i}}{SP_{i}}$$

where

*SP_f_*: the solution pH after the samples soaked for 6 h;
*SP_i_*: the initial solution pH soaked with samples.

#### 2.3.2. The Oil Absorption Test

The oil absorption was evaluated according to ISO787-5-1980 to analyze the compatibility between the coated persistent phosphors and organic solvents [26]. The refined linseed oil (acid value: 5–7 mg/g) was gradually added into the 10 g luminescent powder, stirred evenly until all powder stacked together. 20–25 min is the maximum acceptable test time to guarantee the accuracy. The oil absorption is indicated by the minimum oil consumption in mass percentage. The lower minimum oil consumption in mass percentage means the better organic compatibility.

#### 2.3.3. Thermogravimetric Analysis

The thermal stability of the persistent phosphors was evaluated by a comprehensive thermogravimetric analyzer (TGA/DSC 3+, METTLER TOLEDO, Zurich, Switzerland) in the range from room temperature to 300 °C under nitrogen atmosphere, and the heating rate was 10 °C/min.

#### 2.3.4. Fluorescence Excitation and Emission Test

The excitation and emission spectra of the persistent phosphors were tested by FLs980 full-featured Steady/Transient Fluorescence Spectrometer (Edinburgh Instruments, Edinburgh, UK). Excitation spectrum was scanned from 250 nm to 500 nm. Emission spectrum was monitored from 350 nm to 700 nm.

#### 2.3.5. X-Ray Diffraction

The crystal phase properties were analyzed by X-ray diffraction (XRD) using Bruker D8 ADVANCE (Bruker AXS, Karlsruhe, Germany) as shown in 2θ scan mode from 10 to 60° (2θ) using Cu Kα radiation, with a step width of 0.02° and a time interval of 0.1 s per step [27].

#### 2.3.6. Scanning Electron Microscopy

Scanning electron microscopy (SEM) (S-4800, Hitachi, Japan) was used to analyze the morphological characteristics of persistent phosphors. The resolution increased from 3 to 10 nm with the test voltage ranging from 3 to 40 kV. The accelerating voltage was 3.0 kV [28].

## 3. Results and Discussions

### 3.1. Moisture Resistance of Silica Shell Coating

#### 3.1.1. Effect of the Coating Reaction Time

**Figure 4:**
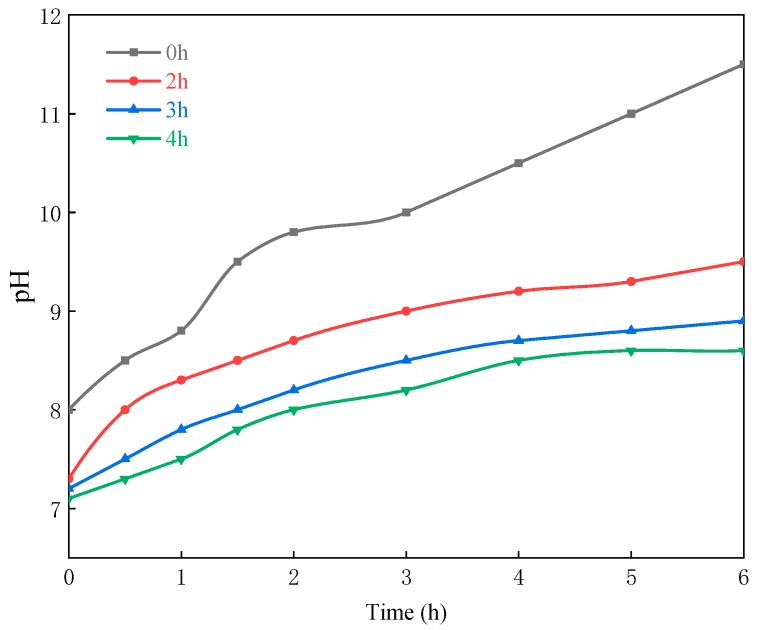
The change of solution pH soaked with PLO-8B luminescent powder coated with different reaction time.

Obviously, the compactness of silica shell increases synchronously with the prolongation of reaction time until the reactions ending, contributing to the improvement of the moisture resistance. However, fully polycondensation reaction is impossible in the coating process due to its resource intensive and time consuming (over 10 h). Thus, three samples of silica shell coating (the coating reaction time is 2, 3, and 4 h respectively) were prepared to determine the optimum time balancing the moisture resistance and preparation time. As shown in Figure 4, the solution pH soaked with PLO-8B luminescent powder coated for 3 and 4 h decreased by 25% and 27.5% compared with the uncoated samples, indicating the moisture resistance of PLO-8B luminescent powder was significantly improved. 3 h was selected as the optimum reaction time for the following tests due to the shorter coating reaction time and similar moisture resistance.

#### 3.1.2. Effect of the Sodium Silicate Dosage

**Figure 5:**
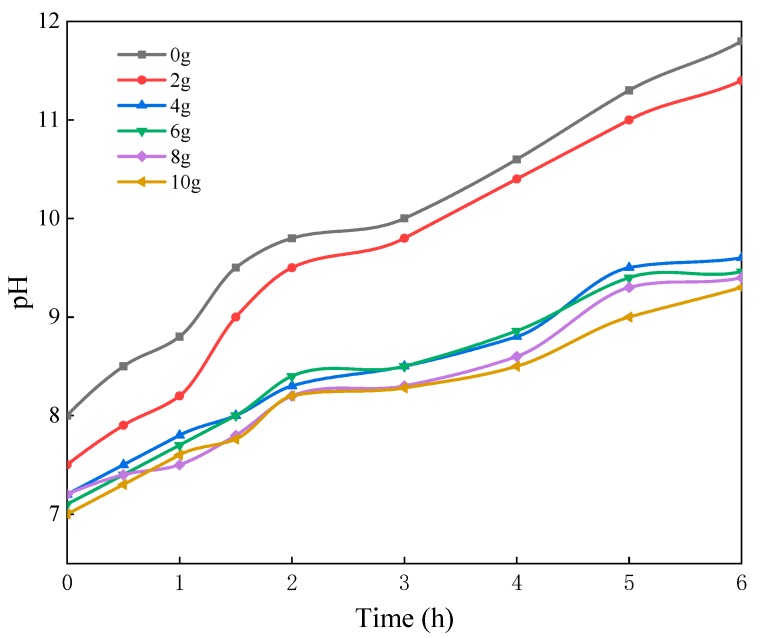
The change of solution pH soaked with PLO-8B luminescent powder coated with different sodium silicate dosages.

SiO_2_·XH_2_O content in Reaction 1 is determined by the sodium silicate dosage resulting in the different thicknesses of the silica shell and coating properties. Five samples of silica shell coating (the sodium silicate dosages are 2, 4, 6, 8, and 10 g respectively) were prepared to determine the optimum dosage. Figure 5 shows the increased sodium silicate dosages decreased the solution pH, indicating effective improvement of the moisture resistance. This improvement was significant as the sodium silicate dosage was less than 4 g, contributing to the 20.34% decreasing compared to the uncoated samples. Further increase of sodium silicate dosages contributed little to improving the moisture resistance of persistent phosphors. Thus, 4 g was selected as the optimum sodium silicate dosage for the following tests.

### 3.2. Moisture Resistance of Polymer Shell Coating

#### 3.2.1. Effect of the Processing Sequence

**Figure 6:**
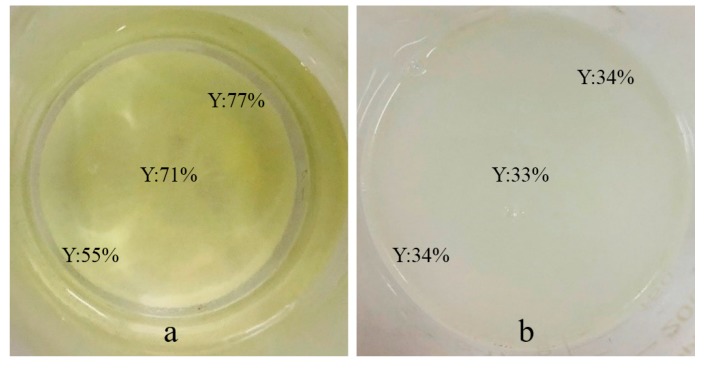
The Y value in hydrolysis solutions of different preparation sequences: (**a**) Add the silane coupling agent first; (**b**) Adjust pH first.

The different preparation sequences of hydrochloric acid and the silane coupling agent from literatures was investigated to achieve better pretreatment. As shown in Reaction 2, the yellow SrAl_2_O_4_: Eu^2+^, Dy^3+^ persistent phosphor is destroyed and turn white after reacting with H_2_O. The degree of hydrolysis reaction was evaluated by the image software based on the value of Y(yellow) in the CMYK(cyan, magenta, yellow, and black) color mode to determine the preparation sequences. Solutions soaked with samples prepared by different sequences was shown in Figure 6. The Y values in the sample with silane coupling agent added first is obvious higher, indicating the better coating for preventing the SrAl_2_O_4_: Eu^2+^, Dy^3+^ persistent phosphor from hydrolysis reaction.

#### 3.2.2. Effect of the Silane Coupling Agent Types

**Figure 7:**
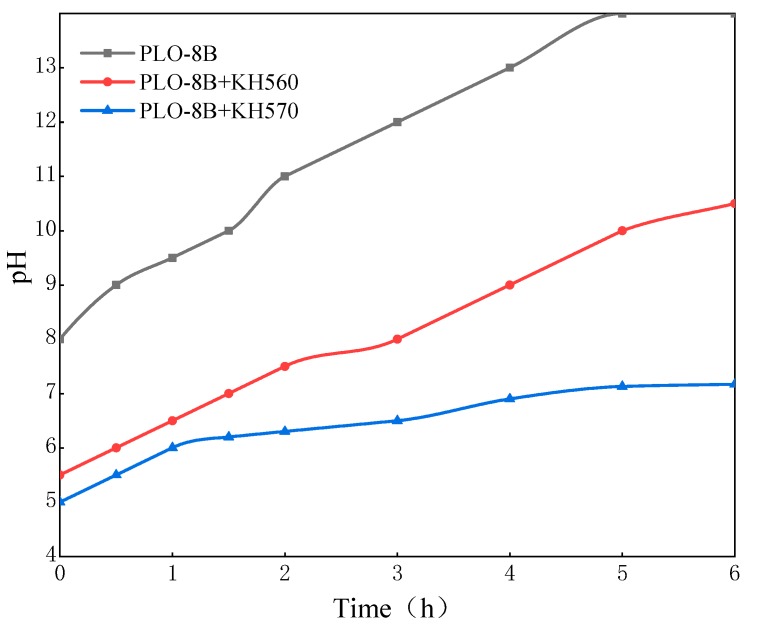
The change of solution pH soaked with PLO-8B luminescent powder coated with different silane coupling agents.

KH560 and KH570 are the mainly silane coupling agents used in the coatings industry. Two silane coupling agents were used to prepare the PLO-8B luminescent powder coated with the polymer shell respectively to determine the optimal type. The moisture resistance was evaluated as shown in Figure 7. The solution was initially acidic due to the hydrogen ions added in the preparation. The untreated PLO-8B luminescent powder hydrolyzed rapidly in 40 °C water resulting in a significant increase in solution pH and almost hydrolyzed completely after 5 h. Two silane coupling agents slowed down the increase of solution pH, indicating the moisture resistance was improved. The solution pH soaked with PLO-8B luminescent powder coated with KH560 and KH570 decreased by 25% and 48.79%, respectively. Obviously, KH570 is more suitable to polymer shell coating for PLO-8B luminescent powder than KH560.

**Figure 8:**
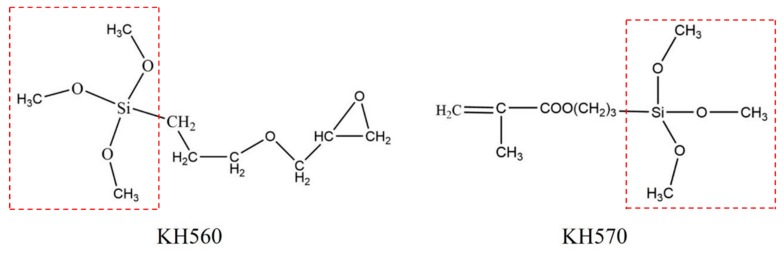
Molecular structures of different silane coupling agents.

Molecular structures of different silane coupling agents are shown in Figure 8. Siloxy groups forming the highly active silanol after the hydrolysis reaction are observed both in the KH560 and KH570, which bonds with the hydroxyl groups on the surface of persistent phosphors and undergo polycondensation by the “Si–OH” functional group. However, the methacrylic group in KH570 is unique. It is copolymerized with MMA and sodium persulfate to generate a denser polymer shell further improving the moisture resistance.

#### 3.2.3. Effect of Silane Coupling Agent Dosages

**Figure 9:**
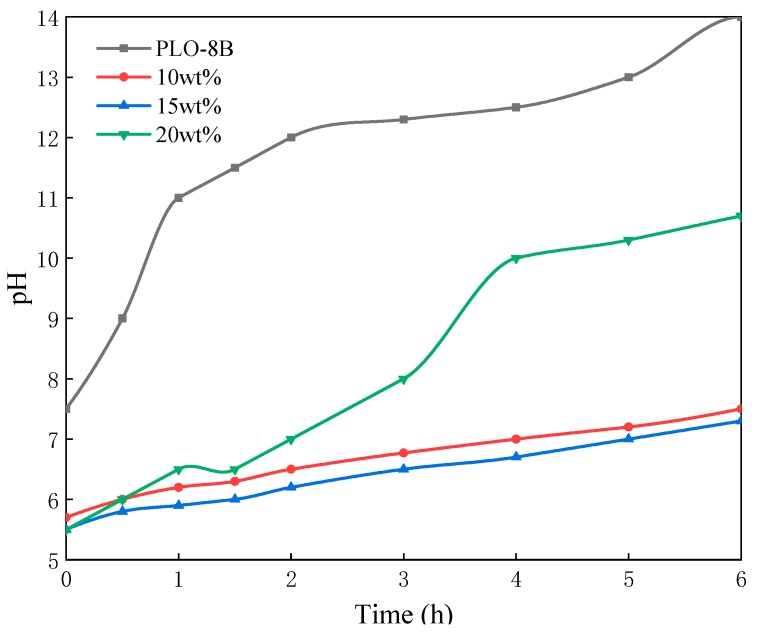
The change of solution pH soaked with PLO-8B luminescent powder coated with different silane coupling agent dosages.

Three samples of polymer shell coating (silane coupling agent dosages are 10, 15, and 20 wt% respectively) were prepared to determine the optimum silane coupling agent dosage. As shown in Figure 9, the solution pH soaked with coated samples decreases clearly. However, the excessive silane coupling agent (20 wt% silane coupling agent) formed the loose and easily broken polymer shell, resulting in the sharp rise of solution pH after the polymer shell broken in 1.5 h. The solution pH soaked with PLO-8B luminescent powder coated with 10 wt% and 15 wt% silane coupling agent decreased by 47.14% compared to the uncoated sample, indicating the significant improvement of moisture resistance. Herein 10 wt% silane coupling agent formed a thinner polymer shell contributing to the less negative impact on the luminescent properties. Thus 10 wt% was selected as the optimum dosage of the silane coupling agent.

#### 3.2.4. Effect of Sodium Dodecyl Benzene Sulfonate Dosages

**Figure 10:**
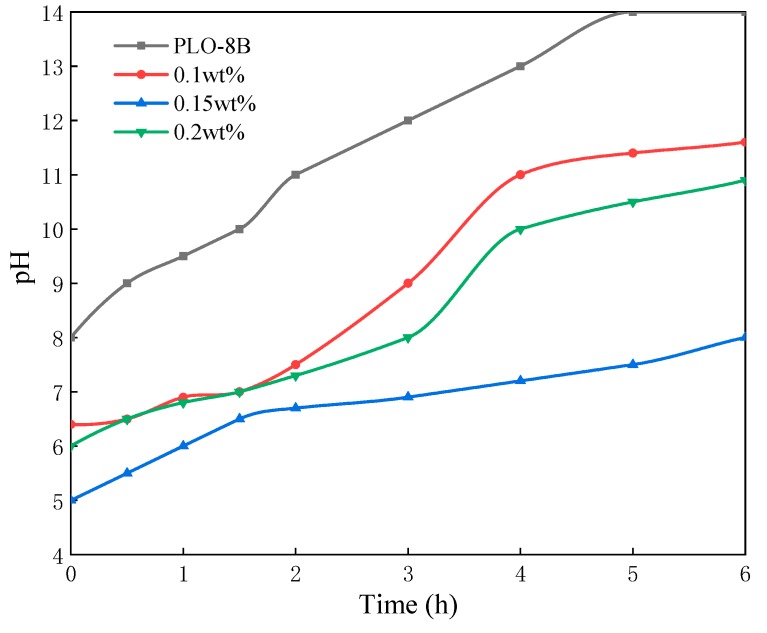
The change of solution pH soaked with PLO-8B luminescent powder coated with different sodium dodecyl benzene sulfonate dosages.

The surfactant (sodium dodecyl benzene sulfonate) dosage determines the conversion rate of the methacrylic monomer, relating to the quality of the polymer shell. Three samples of polymer shell coating (sodium dodecyl benzene sulfonate dosages are 0.1, 0.15, 0.2 wt%, respectively) were prepared to select the optimum surfactant dosage. As Figure 10 shows, the solution pH started significantly increasing from 1.5 h, indicating the polymer shell had been severely damaged. Obviously, the moisture resistance of the polymer shell coating samples with 0.1 or 0.2 wt% surfactant was much worse than 0.15 wt%. Too much surfactant (0.2 wt%) leads to the violent polymerization reaction. The newly grown polymer shell rapidly aggregates on the surface of PLO-8B luminescent powder, resulting in the loose structure of the polymer shell. On the contrary, too less surfactant (0.1 wt%) limits the formation of polymer shell. Thus, 0.15 wt% is selected as the optimum surfactant dosage.

### 3.3. Moisture Resistance of Silica-Polymer Hybrid Shell Coating

**Figure 11:**
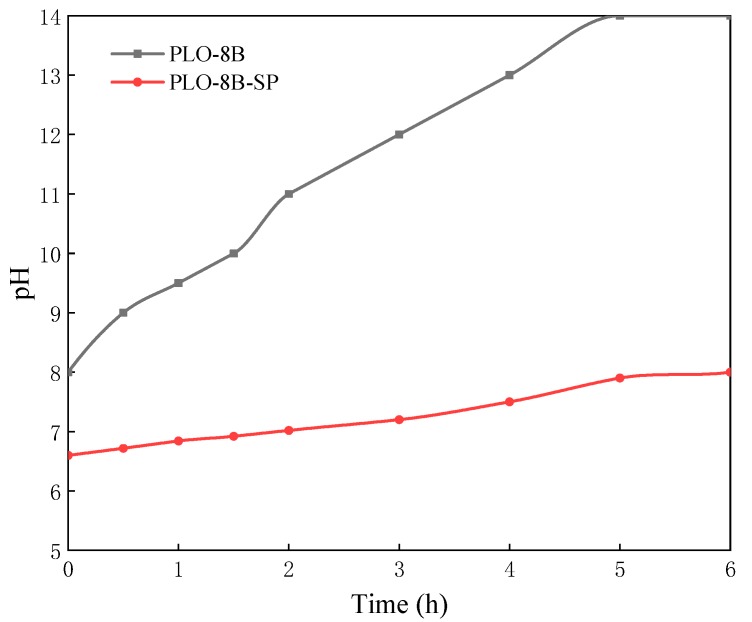
The change of solution pH soaked with PLO-8B luminescent powder coated with silica–polymer hybrid shell.

**Figure 12:**
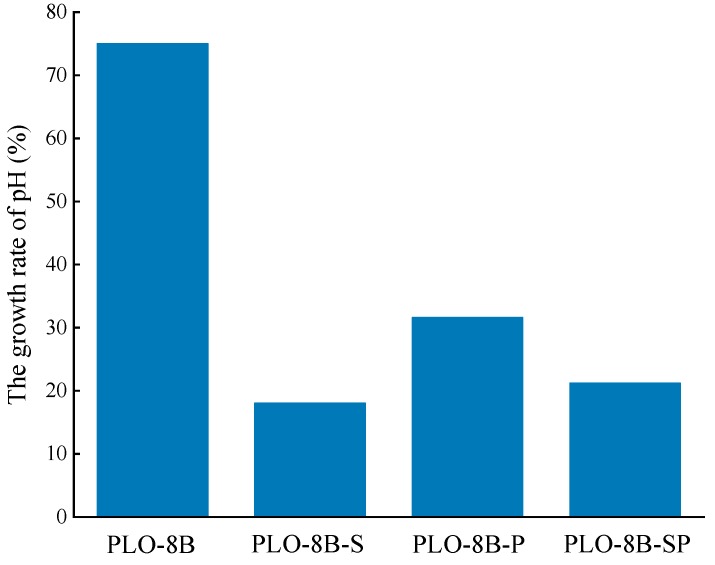
The growth rate of solution pH soaked with PLO-8B luminescent powder coated with silica shell, polymer shell and silica–polymer hybrid shell.

PLO-8B luminescent powder coated with silica–polymer hybrid shell (PLO-8B-SP) was prepared to compare the moisture resistance with PLO-8B luminescent powder coated with silica shell (PLO-8B-S) and polymer shell (PLO-8B-P). As Figure 11 shows, the silica–polymer hybrid shell coating significantly slowed down the increase of solution pH, indicating the improvement in the moisture resistance. The growth rate of solution pH soaked with PLO-8B, PLO-8B-S, PLO-8B-P, and PLO-8B-SP was 75%, 18%, 32%, and 21%, respectively as shown in Figure 12. The PLO-8B-S and PLO-8B-P was prepared based the on the optimal composition from the previous tests. Obviously, all types of shell coating significantly improve the moisture resistance of PLO-8B luminescent powder. The growth rate of solution pH soaked with PLO-8B-S, PLO-8B-P, and PLO-8B-SP was decreased by 75.93%, 57.89%, and 71.72%, respectively compared to PLO-8B. The moisture resistance of PLO-8B luminescent powder coated with silica–polymer hybrid shell was significantly improved compared to polymer shell coating, indicating the silica shell coating contributes the most in the test.

### 3.4. Organic Compatibility of Persistent Phosphors

**Figure 13:**
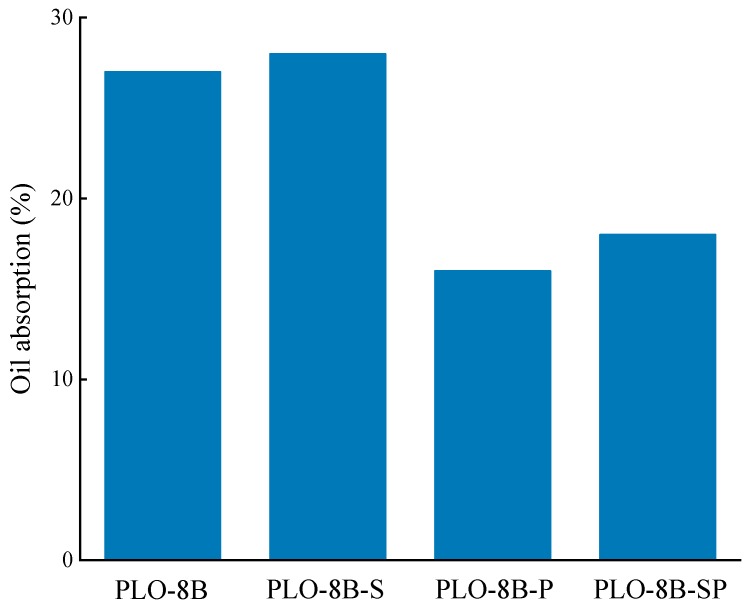
Oil absorption of PLO-8B luminescent powder.

The oil absorption of persistent phosphors shows the organic compatibility, indicating the compatibility with other organic solutions in waterborne coatings such as auxiliaries and binders [29,30,31]. Four samples were prepared to evaluate the organic compatibility based on the oil absorption test according to the ISO787-5-1980 as shown in Figure 13. The organic compatibility of PLO-8B-S, PLO-8B-P, and PLO-8B-SP was improved by 7.41%, 40.74%, and 33.33%, respectively indicating the silica shell coating has little effect on the oil absorption. Both the polymer shell coating and the silica–polymer hybrid shell coating greatly improved the organic compatibility of PLO-8B luminescent powder. Due to the decrease in silane coupling agent and AA/MMA dosage, the organic compatibility improvement of PLO-8B luminescent powder coated with silica–polymer hybrid shell is slightly weaker than that of PLO-8B luminescent powder coated with polymer shell.

### 3.5. Thermal Stability of Persistent Phosphors

**Figure 14:**
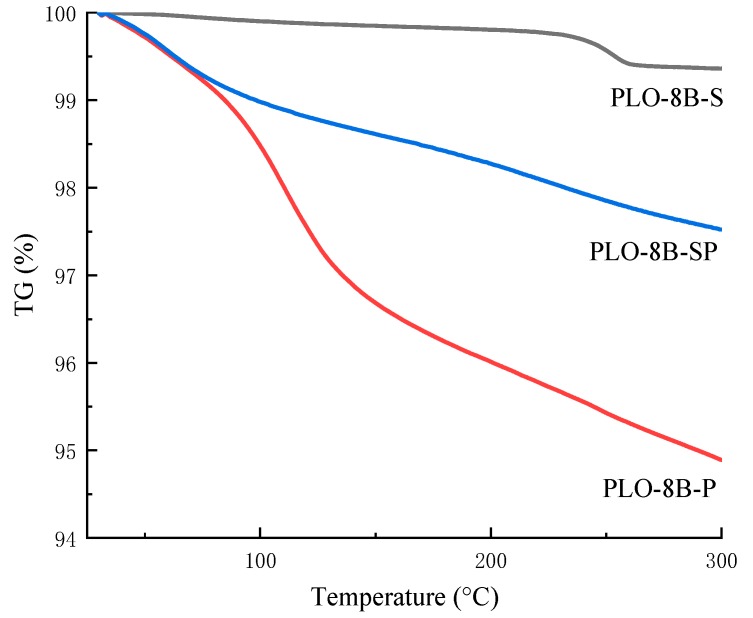
TG curves of PLO-8B luminescent powder coated with silica shell, polymer shell, and silica–polymer hybrid shell.

The comprehensive thermogravimetric analyzer (TGA/DSC 3+, METTLER TOLEDO) was used to measure the TG curves of PLO-8B luminescent powder coated with silica shell, polymer shell, and silica–polymer hybrid shell as shown in Figure 14. PLO-8B-P constantly lost mass with the temperature increasing due to the volatilization of polymer shell. The mass loss of PlO-8B-S was within 0.5%, indicating the better thermal stability. The initial thermal weight loss behavior of PLO-8B-SP was consistent with PLO-8B-P, due to the same polymer shell. However, the less silane coupling agent and AA/MMA dosage formed the thinner polymer shell coating the PLO-8B luminescent powder in PLO-8B-SP. Thus, the thermal weight loss of PLO-8B-SP slowed down gradually. Obvious, the easily decomposable polymer shell coating is not suitable to prepare the dedicated aluminate-based persistent phosphors for the durable luminescent road surface marking, due to the extremely high temperatures of pavement surface in some cases. The silica and silica–polymer hybrid shell contributed to the better thermal stability of PLO-8B-S and PLO-8B-SP, indicating the ability to ensure the moisture resistance in the wider range of temperature.

### 3.6. Luminous Performance of Persistent Phosphors

**Figure 15:**
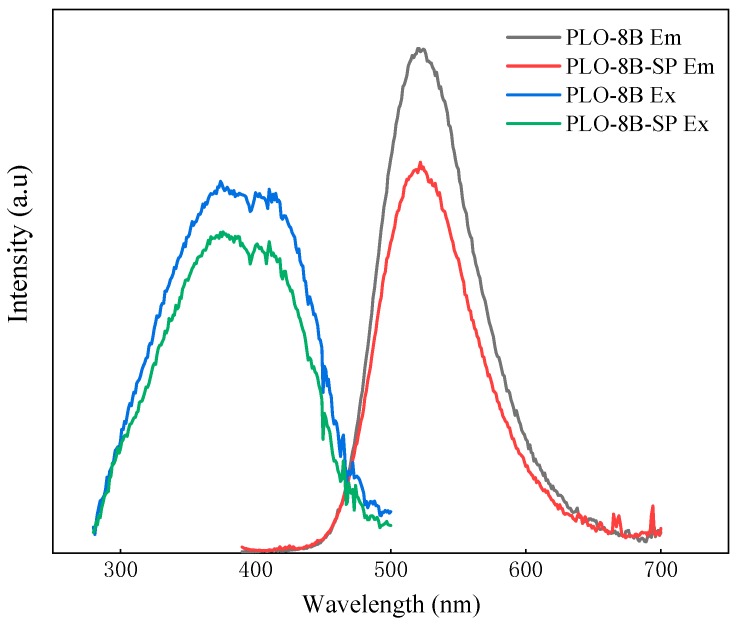
Em and Ex spectra of PLO-8B and PLO-8B-IO.

FLs980 full-featured Steady/Transient Fluorescence Spectrometer (Edinburgh) was used to measure the excitation and emission spectra of PLO-8B luminescent powder (PLO-8B) and PLO-8B luminescent powder coated with silica–polymer hybrid shell (PLO-8B-SP). The luminescence properties were analyzed by comparing the spectral peaks and peak areas. According to Figure 15, the peak positions of PLO-8B-SP in the excitation spectrum and the emission spectrum are consistent with the PLO-8B, while the peak decreased. Due to part of light was reflected or absorbed by the silica–polymer hybrid shell, the persistent phosphors absorbed less light energy, resulting in the attenuate of emitted light. The peak emission spectrum and excitation spectrum of the PLO-8B-SP decreased by 23.4% and 13.7%, respectively. Obviously, the silica–polymer hybrid shell coating affects the brightness but does not damage the persistent phosphors.

### 3.7. X-Ray Diffraction (XRD) Analysis

**Figure 16:**
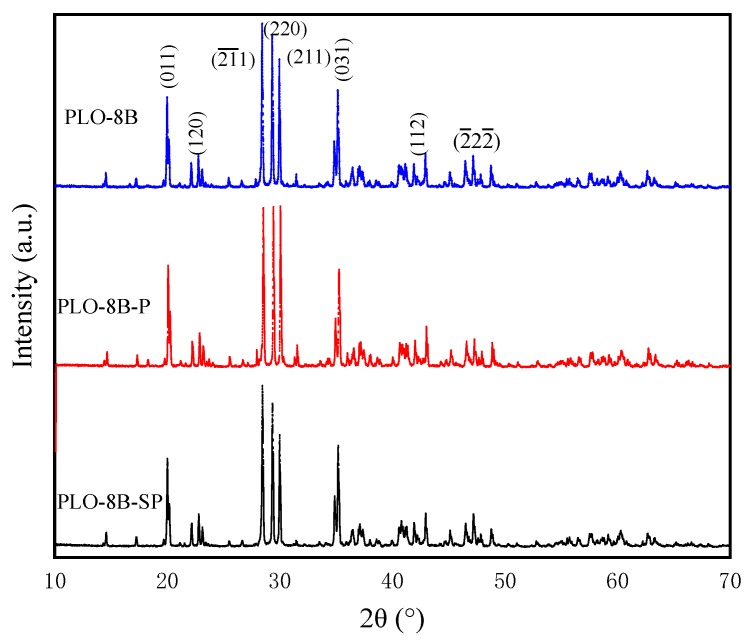
XRD patterns of PLO-8B luminescent powder (PLO-8B), PLO-8B luminescent powder coated with polymer shell (PLO-8B-P), and PLO-8B luminescent powder coated with silica–polymer hybrid shell (PLO-8B-SP) [32].

Figure 16 plots XRD patterns of PLO-8B luminescent powder (PLO-8B), PLO-8B luminescent powder coated with polymer shell (PLO-8B-P), and PLO-8B luminescent powder coated with silica–polymer hybrid shell (PLO-8B-SP). All the diffraction peaks were indexed and are good in agreement with JCPDS card no. 034-0379 with lattice constants a = 8.4424Å, b = 8.822Å, c = 5.1607 Å with interfacial angles α = 90.00°, β = 93.42°, γ = 90.00°, respectively. Hence persistent phosphors of were not changed, which is consistent with the results of Fluorescence excitation and emission test.

### 3.8. Scanning Electron Microscopy (SEM) Analysis

**Figure 17:**
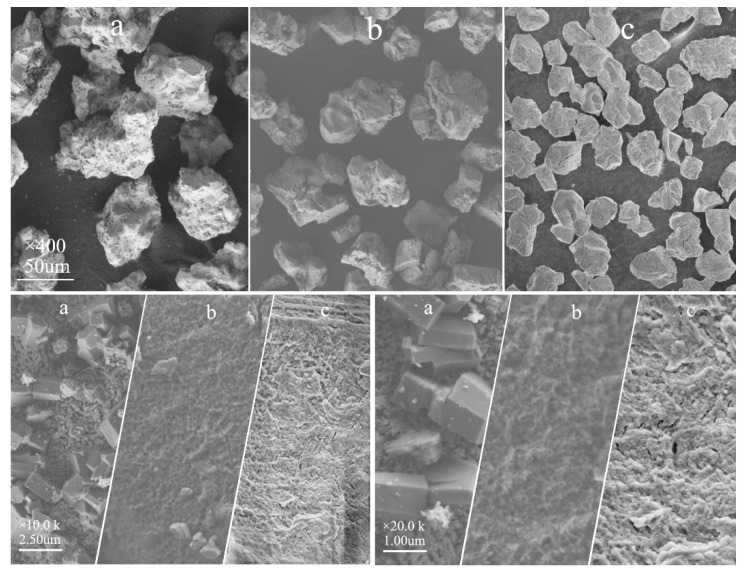
SEM images: (**a**) PLO-8B; (**b**) PLO-8B-P; (**c**) PLO-8B-SP.

The SEM images of samples are shown in Figure 17. As can be seen under 400× magnification, the surface irregularity of PLO-8B is weakened after coated with shells, which means the surface covered by a complete protective layer. Due to the addition of silica shell, the surface of PLO-8B-SP is smoother than PLO-8B-P. As can be seen under 10,000× magnification, excess silane coupling agent reacted with AA/MMA to form a small amount of protrusion on the surface of PLO-8B-P. A dense and intact protective layer appeared on the surface of PLO-8B-IO, indicating the reduction of silane coupling agent and AA/MMA dosage is suitable. As seen under 20,000× magnification, the coated shells form a honeycomb structure covering the regular and distinct crystal structure of PLO-8B, which protects the luminescent structure from damage.

## 4. Conclusions

SrAl_2_O_4_: Eu^2+^, Dy^3+^ persistent phosphors coated different type of shells were prepared in this paper to improve moisture resistance and organic compatibility. The optimal parameters (coating reaction time, sodium silicate dosage, processing sequence, silane coupling agent types, silane coupling agent dosages, and sodium dodecyl benzene sulfonate dosages) of chemical precipitation and sol-gel method for the preparation of silica–polymer hybrid shell were determined by the moisture resistance test. The silica–polymer hybrid shell coating balances the organic compatibility and thermal stability as compared to the silica or polymer shell coating in the oil absorption test and thermogravimetric analysis. The phase structure of SrAl_2_O_4_: Eu^2+^, Dy^3+^ persistent phosphors did not change in the coating process based on luminous performance, XRD, and SEM analysis. The optimal silica–polymer hybrid shell coating for SrAl_2_O_4_: Eu^2+^, Dy^3+^ persistent phosphors improved the moisture resistance and organic compatibility by 71.72% and 33.33% compared to the uncoated SrAl_2_O_4_: Eu^2+^, Dy^3+^ persistent phosphors. The outstanding durable properties of SrAl_2_O_4_: Eu^2+^, Dy^3+^ persistent phosphors coated with silica–polymer hybrid shell as shown in this research is crucial for its potential application in waterborne luminescent coatings of road surface marking.

## Figures and Tables

**Table 1:** Dosages of the coating with silica shell.

Reagent	Weight
PLO-8B luminescent powder (PLP)	5 g
Ethylene glycol	50 g
Sodium silicate	4 g

**Table 2:** Dosages of the coating with polymer shell.

Reagent	Weight
PLO-8B luminescent powder (PLP)	10 g
Silane coupling agent	1 g
Anhydrous ethanol	70 g
Acrylic acid monomer (AA)	0.5 g
Methyl methacrylate (MMA)	0.5 g
Sodium persulfate	1 g
Sodium dodecyl benzene sulfonate	0.15 g
Water	30 g

**Table 3:** Material dosages of the coating of silica–polymer hybrid shell.

Step	Reagent	Weight
Inorganic coating	PLO-8B luminescent powder (PLP)	5 g
	Ethylene glycol	50 g
	Sodium silicate	4 g
Organic coating	Silane coupling agent	0.2 g
	Anhydrous ethanol	70 g
	Acrylic acid monomer (AA)	0.1 g
	Methyl methacrylate (MMA)	0.1 g
	Sodium persulfate	1 g
	Sodium dodecyl benzene sulfonate	0.15 g
	Water	30 g
